# A quantitative study on the impact of working environment on the well-being of teachers in China’s private colleges

**DOI:** 10.1038/s41598-022-07246-9

**Published:** 2022-03-01

**Authors:** Jinping Chen, Hongyan Cheng, Dan Zhao, Fuyu Zhou, Yuan Chen

**Affiliations:** 1 School of Education, Central China Normal University, Wuhan, 430079 China; 2 School of Foreign Languages, Wuhan College, Wuhan, 430212 China; 3 Department of Foreign Languages, Wuhan Institute of Design and Sciences, Wuhan, 430205 China

**Keywords:** Psychology, Environmental social sciences

## Abstract

Teacher well-being is a prominent issue in policy and public debates. Those teachers working in private schools deserve concern given concerns about their working environment. Focus of this study: to learn about the level and main characteristics of teacher well-being in private schools and to explore the impact of working environment on their well-being. Methodology: The data were collected via an online questionnaire among 1161 language teachers at 58 China’s private colleges in 22 provinces, and were quantitatively analyzed with SPSS 23.0. Findings: These teachers have an intermediate level of overall well-being. Performance evaluation, student academic foundation, and pressure of research work are the main negative impacting factors; while social support from leaders and colleagues, work autonomy, training and development opportunities, and appraisal feedback guide are key resources enhancing their well-being. Discussions: The impacting factors of working environment on teacher well-being in private schools are unique due to the special scenarios. Implications: The results of this study may apply to similar private schools, suggesting pertinent stakeholders to take targeted measures, to guarantee enough economic input into running school, and to put democratic and professional leadership into practice for promoting teacher well-being in the private education sector.

## Introduction

In the current context of teaching profession characterized by high levels of burnout and teacher attrition, teachers’ well-being has become a prominent issue in policy and public debates. It is widely suggested that teachers’ well-being plays a critical role in their abilities to teach effectively and creatively in classrooms^1,2^, to build good relationships with students, to enhance student achievement and well-being^3–6^, and to promote the whole school effectiveness^6^. Hence, to improve teacher well-being should be a primary objective of educational leaders. In terms of well-being, teachers working in the private sector suffer additional difficulties compared with those working in public schools, like worse working conditions^7^, less opportunities for mid-career studies and research training^8^, and even low respect from students^9^. Their well-being is defined by the business model character of the private sector particularly in terms of working conditions^10^, so it deserves more concerns. However, existing relevant studies are mainly about the well-being of teachers in public schools, and most studies on this topic regard teacher well-being as a single concept or focus on teachers’ subjective well-being. Furthermore, the samples of most research on this topic come from European countries, the United States and Australia; but the working environment of teachers varies from countries to countries and from school types to school types. Until now, there are few publications to investigate teacher well-being in private schools, which account for a unique and important part of educational system. Hence, there is still a gap of knowledge about teacher well-being, especially those in private schools and those from different cultures and countries. Teacher well-being is a complex concept consisting of different dimensions, and is impacted with a wide range of factors which are different in different types of schools and in different countries. More studies are needed to know more about teacher well-being in private schools, its shaping factors and promoting measures, as types of privatization of education and the roles of private schools vary from country to country. This study aims to contribute to the literature via exploring the impacting factors of teacher well-being in private schools in China with the multi-dimensional concept of teacher well-being, and to provide evidence-based directions for practice. The central research question is “what is the unique working environment in terms of job demands and job resources impacting the well-being of teachers in private schools”, which will be addressed via an online questionnaire survey and quantitative statistical analyses.

Generally speaking, teacher well-being is impacted with a wide array of factors of four kinds, i.e. the individual (i.g. character, resilience, adaptability, and self-efficacy)^11,12^, organizational (i.g. school type, school size, school location, and **working environment**)^13,14^, institutional (i.g. earnings, labor market security, and career structure)^15,16^, social and cultural (i.g. expectations, values, and legislation)^17^. Recently, more and more studies highlight the quality of the working environment and identify it as a major driver of teachers’ well-being and an important predictor of teachers’ job satisfaction^18–21^. A study based on American national data finds that school environment exerts substantial influence on teachers’ job satisfaction^22^, and empirical evidence from private universities in Bangladesh also concludes that working environment in schools is a determinant of teachers’ job satisfaction^7^. Hence, to take intervention measures to promote teacher well-being, school working environment has become the focus of concerns.

To analyze the working environment at the school level, job demands-resources (JD-R) theory, which emerged in recent years as one of the most influential conceptual frameworks for interpreting and explaining factors affecting employees’ well-being in the workplace, provides an effective theoretical framework. One of the strengths of the JD-R theory is its ability to link key contextual factors to well-being in a dual process model. In this theory, job demands are the physical, social or organizational aspects of the job that entail physical and/or psychological costs from teachers^13^, while job resources are conversely the physical, psychological, social or organizational aspects of the job that stimulate personal growth, learning and development, and help achieve work goals and reduce job demands^23^. Employed for research on teacher well-being, this JD-R model, by comparing the impact of job demands and job resources, allows for insights into how teacher well-being is impacted at organizational level and for analysis of the guiding interventions to maximize teacher well-being. Existing research about teacher well-being has identified some significant indicators for job demands and job resources. For job demands, they are workload^24^, which measured mainly through yearly teaching hours and research work (especially for college teachers)^25^, time pressure, which is usually measured with daily working hours^26^, classroom composition, which is reflected in class size and student differences^27^, and performance evaluation^28^; For job resources, they are work autonomy^29,30^, development opportunities^26^, appraisal feedback guide^23,24,30^, social support from leaders and colleagues^21,24^.

To explore the impact of working environment on teacher well-being in private schools, this study makes a survey about the level and main characteristics of their well-being, and the perceived working environment in two aspects of job demands and job resources in private schools, and then makes quantitative analyses. For the general design of a quantitative survey study, theoretical hypotheses will firstly be drawn from past research, applied questions or theories. According to the JD-R model of working environment, we hypothesize that job demands serve as negative factors and job resources as positive factors. More specifically, based on above-mentioned existing literature about the significant job demands and job resources impacting teacher well-being, we hypothesize that: (H1) heavier teaching and research workload and time pressure, more students and lower level of base knowledge in a class, and more stressful performance evaluation decrease teacher well-being; (H2) more work autonomy, satisfying development opportunities and helpful appraisal feedback guide, and stronger social support from leaders and colleagues enhance teacher well-being. The quantitative analyses will validate the hypotheses and identify the impacting factors of working environment on teacher well-being in private schools. As for the conceptual framework of teacher well-being, the Organization for Economic Co-operation and Development (OECD) defines it in four dimensions of cognitive well-being, subjective well-being, physical/mental well-being, and social well-being^31^, so we will consider teacher well-being both in overall well-being and in the four dimensions as well in order to make an in-depth exploration of teacher well-being in private schools and its impacting factors in terms of working environment.

## Methods

For the general design of a quantitative survey study, theoretical hypotheses will firstly be drawn, and then a survey will usually be conducted with appropriate samples and measurement instruments, and then the hypotheses will be validated through quantitative statistical analyses with the data. For this specific study, two aforementioned hypotheses have been created; then a survey is conducted online with convenient sampling in China’s private colleges and the measurement instruments by OECD; then the two hypotheses are validated with data to find the unique impacting factors of working environment on the well-being of teachers in private schools, with discussions of the results in the end.

### Participants

In this study, online survey and convenient sampling are used to select the participants, which are language teachers in China’s private colleges. The selection method is as follows. To conduct the survey, we firstly designed the questionnaire on a popular online survey website called *Questionnaire Star* (https://www.wjx.cn/) in China to get a link for access. We also set a password for entry to avoid hacker visits. Then we began to contact deans of foreign language college/department at private colleges across the country. The first author of this study, also a dean at a private college for many years, got to know many other deans at past national conferences. With help from various sources, he sent the link to 60 schools and got active response from 58, which are located in 22 provinces/municipalities across mainland China. The 58 deans then forwarded the link to their teachers in their working QQ group or Wechat group. Teachers filled in the questionnaire on the smartphone, PC or tablet voluntarily and anonymously. The survey was conducted during January of 2021 before the end of the school semester. Finally, we collected 1161 questionnaires from full-time teachers affiliated only to private colleges, which are all complete and valid. Table 1 lists the teachers’ profiles. As can be seen, this group of teachers are primarily females (83.81%), with education degree of master (83.98%), academic titles of lecturer (53.14%), associate professor (24.46%) and assistant (20.67%), and teaching years below 20 (95.01%). The sample features are very close to those descriptions in previous literature^8,32,33^. The steps of selecting participants accord with the requirements of quantitative study, and the demographic proportions of participants can stand for the whole body as described in previous surveys; hence the participants in this study are reasonable and acceptable.

**Table 1:** Respondents’ profiles (*n* = 1161).

Item	Category	Percentage (%)	Item	Category	Percentage (%)
Gender	Female	83.81	Academic title	Assistant	20.67
	Male	16.19		Lecturer	53.14
Age	20–30	19.72		Associate Prof	24.46
	31–40	62.10		Professor	1.72
	41–54	16.71	Teaching years	≤ 5	25.67
	≥ 55	1.47		6–10	26.36
Education degree	Junior college	1.46		11–20	42.98
	Bachelor	12.32		21–30	3.70
	Master	83.98		≥ 31	1.29
	Doctor	2.24			

### Instruments

We adopt the questionnaire of “PISA2021-teacher” offered by OECD to measure teacher well-being and working environment in terms of job demands and job resources in private schools. Several questions about the participants’ demographics are also included in the questionnaire as reported in Table 1.

#### Measurement of demographics

We design 6 questions by ourselves and participants are asked to select gender from male or female, select age from “20–30, 31–40, 41–54, ≥ 55”, select education degree from “Junior college, Bachelor, Master, Doctor”, select academic title from “Assistant, Lecturer, Associate Prof., Professor”, select teaching years from “ ≤ 5, 6–10, 11–20, 21–30, ≥ 31”.

#### Measurement of self-perceived sense of well-being

The conceptual framework of teacher well-being proposed by OECD^31^ summarizes various aspects involved in teachers’ occupational well-being and the ways in which these aspects interconnect. The framework defines teacher well-being around four key dimensions: cognitive, subjective, physical/mental, and social. The indicators to measure cognitive well-being include teachers’ capacity to concentrate on work (how often the teachers experienced mindfulness in the past two weeks) and self-efficacy in classroom management, in instruction, and in student engagement (to what extent teachers can do well); those indicators for subjective well-being include teachers’ job satisfaction (how teachers generally feel about their profession and working conditions), life satisfaction (how satisfied teachers are with their life), affects (mood and state of mind in the past two weeks), and purposefulness (sense of direction and purpose, making working plans for the future); psychosomatic symptoms are mainly measured to indicate physical/mental well-being (insomnia, fatigue, headache, dizziness, anxiety, irritability, feeling down, etc.); and for social well-being, teachers’ relationships with colleagues, leaders, students and their feeling of trust are considered. The overall well-being was an aggregate of the four dimensional well-beings.

#### Measurement of self-perceived job demands and job resources

The OECD framework also provides questionnaires to measure the quality of the working environment at the school level. According to the aforementioned hypotheses in this study, we take such indicators for job demands: yearly teaching hours, pressure for research work, daily working hours, class size (the number of students in a class), student academic foundation (teachers’ perceived knowledge base of students), and performance evaluation (school evaluations on teachers’ performance and achievements); while such indicators for job resources: work autonomy (perceived how much), training and development opportunities (how satisfied and how helpful), appraisal feedback (whether give feedback on the evaluations and how helpful), and social support from leaders and colleagues (perceptions of relationships, recognition, and helpfulness).

A pilot survey with the draft questionnaire was conducted in December of 2020 among 297 language teachers in 12 China’s private colleges. After clearly rewording some vague items and deleting some items based on the scale analysis with ‘Cronbach α value if deleted’, a final questionnaire was constructed with four sections. Section one includes respondents’ demographic items such as gender, age, seniority, education background. Section two is the main part of this questionnaire survey, including items measuring the four dimensions. The third section examines the impacting factors in terms of job demands and job resources. Section four contains statements on teachers’ perceived stress / burnout and motivation to leave teaching. Items in sections two to four are mostly coded on 5-point Likert scales where 1 = strongly disagree, 2 = disagree, 3 = fair, 4 = agree and 5 = strongly agree, while items related to frequencies are coded on 5 scales from low frequency to high, and some single items like working hours (select one from “5,6,7,8,9,10,11,≧12”), class size (select one from “≦30, 31–50, 51–100, 101–150, 151–200, > 200”), appraisal feedback (select one from “no feedback, feedback with score and ranking, feedback with score and ranking and improvement advice”), perceived work stress (select one from 0 to 10), motivation to leave teaching (yes or no to leave teaching profession) are tested exclusively.

With the use of SPSS 23.0, we analyzed the internal consistency coefficient of the final well-being scales. The Cronbach Alpha value of the overall well-being scale is 0.86 (33 items) with the Alpha value for cognitive well-being being 0.70 (9 items), for subjective well-being 0.88 (8 items), for physical/mental well-being 0.86 (5 items), and for social well-being 0.91 (11 items). Generally speaking, scale with Cronbach Alpha value above 0.70 means sound internal consistency. According to the reliability analysis results in this research, all the Alpha values exceed the criteria 0.70, implying good and acceptable reliability of the scales.

As for validity, this study used the teachers’ well-being framework and scales reported by OECD with reasonably sound content validity. We used exploratory factor analysis to measure the construct validity of this instrument. And it is found that: KMO values of the overall well-being, cognitive well-being, subjective well-being, physical/mental well-being and social well-being scales are respectively 0.86, 0.86, 0.87, 0.82, 0.90, and all the Bartlett’s Test of Sphericity are statistically significant with *P* < 0.001. Confirmatory factor analysis was then applied to measure the validity of the scales with AMOS 24.0, and all the four measurement models have been tested acceptable with all the statistically significant Critical Ratio values (*P* < 0.01), and all the path coefficient values bigger than 0.50. Finally, according to the usual way measuring validity of scale^34^, we calculated values of AVE (Average Variance Extracted) and CR (Composite Reliability) with the path coefficient values. It is found that values of AVE for the four dimensions of teachers’ well-being are respectively 0.50, 0.53, 0.55 and 0.50 (all equal or above 0.5), while the values of CR are respectively 0.84, 0.90, 0.86, 0.91 (all above 0.7). It indicates sound convergence and construct validity of the well-being scales.

### Statistical analysis

At first, we use SPSS 23.0 to perform descriptive statistical analyses to get the mean, standard deviation of the overall and four dimensions of teacher well-being to explore the level and main characteristics of teacher well-being.

Then, independent samples t-test and one-way ANOVA are undertaken to examine whether there are significant demographic group differences of self-reported well-being due to the tested homogeneity of variance and normality of the distribution of aggregate variables of well-being dimensions. Demographic variables like age, education level, etc. are included as independent variables, while aggregate overall well-being, each well-being dimension are included into the T test and ANOVA analysis as dependent variables.

For the impacting factors, we use multiple linear regression analyses to explore the determinants of teachers’ aggregate overall well-being and the four dimensions, which are respectively put into the multivariate regression model as dependent variables. Variables on job demands and job resources are included as independents ‘entering’ rather than stepwise, backward, forward or remove, into the assumed linear regression models. Model fit and Collinearity diagnostics are ticked as statistics, while Standardized and Unstandardized are selected in the Predicted Values group, and Standardized in the Residuals group. By comparing the unstandardized coefficient (*B* and *SE*), standard coefficient (*Beta*), and *t* and *p* values, we try to find out the impacting factors and how strong they are.

### Ethics

This study was approved by the Ethics Committee of School of Education at Central China Normal University. All of the procedures were performed in accordance with the Declaration of Helsinki and relevant policies in China. All participants agreed to participate voluntarily, with informed consent when they filled in the survey and were able to withdraw from the study freely at any time. The questionnaire was designed and applied to ensure anonymity of participants. The data were confidential and participation was anonymous without any potential risk to the integrity of the subjects.

## Findings

The purpose of this study is to find the impacting factors of working environment in terms of job demands and job resources on the well-being of teachers in private schools. Before coming to that, we first examine the level of main characteristics of their well-being by analyzing the mean values and standard deviation of the overall well-being and the four dimensions, and significant differences between different demographic teacher groups in regard to overall well-being and the four dimensions. Then, we address the core question of impacting factors by multiple linear regression analyses of job demands and job resources on overall well-being and the four dimensions, in order to find how job demands and resources might be associated with overall well-being and different facets of well-being in different ways.

### Level and main characteristics of well-being

Table 2 shows that language teachers in China’s private colleges have an intermediate level of overall well-being with the mean value of 3.67 and the lowest *SD* value. Among the four dimensions, they have the highest cognitive well-being, but the lowest physical/mental well-being (converse re-coding the original data from 5 to 1 for analyses), and they also differ greatly in physical/mental dimension with the highest *SD* value, which means some of the teachers are suffering relatively severe psychosomatic problems like fatigue, headache, insomnia, and dizziness. Their subjective well-being and social well-being stand in the middle among the four.

**Table 2:** Descriptive analysis results of teachers’ well-being (*n* = 1161).

	Overall WB	Cognitive WB	Subjective WB	Physical/mental WB	Social WB
Mean	3.67	4.13	3.44	3.10	3.71
SD	.50	.60	.71	.88	.61

As for different demographic groups of teachers, they differ in overall well-being or in some dimensions. Female teachers are significantly different from males only in cognitive dimension (*t* = 2.22, *p* = 0.03), and they have higher cognitive well-being (*M* = 4.15 vs 4.04). For teachers of different ages, they are different in overall well-being (*f* = 6.99, *p* < 0.01), subjective well-being (*f* = 9.06, *p* < 0.01), physical/mental well-being (*f* = 5.60, *p* < 0.01), and social well-being (*f* = 4.98, *p* < 0.01), but not different in cognitive well-being (*f* = 0.89, *p* = 0.44). Teachers above 55 years enjoy the highest well-being, then those younger teachers of 20–30, then those 41–55, and finally those 31–40, with respect to overall well-being, subjective well-being, physical/mental well-being, and social well-being. Similarly, teaching years have the same diachronic correlations with teacher well-being as the ages. Teachers with longest teaching years (≥ 30) and shortest teaching years (≤ 5) enjoy higher overall well-being, subjective well-being, physical/mental well-being and social well-being than those with 21–30, 6–10, and 11–20 years. Teachers with different education degrees differ only in subjective well-being (*f* = 5.86, *p* < 0.01) and social well-being (*f* = 2.80, *p* = 0.04), with the high-to-low order for subjective well-being as those with junior college certificate, bachelor degree, master degree, and doctor degree, and with high-to-low order for social well-being as those with junior college certificate, bachelor degree, doctor degree, and master degree (only one place different from before). Teachers with different academic titles are significantly different in overall well-being (*f* = 11.60, *p* < 0.01), subjective well-being (*f* = 10.27, *p* < 0.01), physical/mental well-being (*f* = 9.57, *p* < 0.01), and social well-being (*f* = 9.78, *p* < 0.01), but not different in cognitive well-being (*f* = 1.30, *p* = 0.27), very much like that in terms of teachers’ age. Professors and assistants enjoy the highest in the four aspects, with associate professors higher than lecturers in overall, subjective, and social well-being, and lecturers higher than associate professors only in physical/mental well-being.

### Impacting factors

It is assumed that impacting factors of teacher well-being come from two categories of job demands and job resources according to the JD-R model. The whole category should not be regarded as a sole impacting factor^35^, so we put every indicator in a category into regression analysis as a single predictor. Through the multiple linear regression analyses, we get bunches of factors for the overall well-being and each dimension, and put those factors of significance (*P* ≤ 0.05) in the following tables in the order of *Beta* value from high to low, which means the influencing degree from high to low.

There are 8 statistically significant factors influencing overall well-being as shown in Table 3. Out of job resources, social support from leaders, social support from colleagues, and work autonomy have strong positive effect, and training and development opportunities, appraisal feedback guide also play significant roles. In respect of job demands, performance evaluation, student academic foundation, and pressure of research work have significant negative impact.

**Table 3:** Impacting factors of overall well-being (*n* = 1161).

		B	SE	Beta	*t*	Sig	Tolerance	VIF
Job resources	Social support-leaders	.24	.02	.38	14.72	< .01	.48	2.09
Job resources	Social support-colleagues	.14	.01	.21	9.74	< .01	.70	1.44
Job resources	Work autonomy	.03	.01	.13	6.41	< .01	.73	1.37
Job demands	Performance evaluation	− .06	.01	− .12	5.82	< .01	.71	1.41
Job resources	Training and development opportunities	.05	.01	.09	3.77	< .01	.50	1.99
Job resources	Appraisal feedback guide	.03	.01	.07	3.31	< .01	.63	1.59
Job demands	Student academic foundation	− .05	.01	− .07	3.39	< .01	.79	1.27
Job demands	Pressure of research work	− .01	.00	− .05	2.74	.01	.83	1.21

R^2^ = .64; F = 155.63, *P* ≤ .05.

In regard to the four dimensions of well-being, the numbers of statistically significant impacting factors vary from 4 to 8 (see Table 4). Multicollinearity is generally examined with Tolerance and Variance Inflation Factor (VIF). The cut of value for Tolerance was > 0.10 and < 10 for VIF. In this study, no multicollinearity has been found in these models. For cognitive well-being, there are 7 significant factors with social support from leaders as the leading one, followed by work autonomy with the same *Beta* value. As for other job resources, appraisal feedback guide and social support from colleagues also have positive impact. Among job demands, yearly teaching hours has positive effect and performance evaluation and student academic foundation have negative effect. There are 8 significant factors influencing subjective well-being, among which job resources (i.e. training and development opportunities, social support from leaders, work autonomy, social support from colleagues, appraisal feedback guide) play important roles. Out of job demands, performance evaluation, student academic foundation, and yearly teaching hours have negative impact. Physical/mental well-being has only 4 significant factors on, with daily working hours, performance evaluation and pressure of research work as three negative ones, followed by training and development opportunities as the only positive factor. There are 7 significant factors influencing social well-being, and social support from leaders and colleagues is extremely important (with the highest *Beta* values of 0.56 and 0.38). Work autonomy and appraisal feedback guide also function positively. As for job demands, student academic foundation has negative effect, while daily working hours and yearly teaching hours play positive roles.

**Table 4:** Impacting factors of four well-being dimensions (*n* = 1161).

		B	SE	Beta	t	Sig	Tolerance	VIF
**Impacting factors of cognitive well-being**								
Job resources	Social support-leaders	.11	.03	.15	3.80	< .01	.48	2.09
Job resources	Work autonomy	.05	.01	.15	4.64	< .01	.73	1.37
Job demands	Yearly teaching hours	.05	.01	.12	4.15	< .01	.89	1.12
Job resources	Appraisal feedback guide	.05	.02	.09	2.74	.01	.63	1.59
Job demands	Performance evaluation	-.05	.02	-.07	2.34	.02	.71	1.41
Job demands	Student academic foundation	-.06	.02	-.07	2.41	.02	.79	1.27
Job resources	Social support-colleagues	.05	.03	.07	2.04	.04	.70	1.44
R^2^ = .18; F = 19.62, *P* ≤ .05.								
**Impacting factors of subjective well-being**								
Job resources	Training and development opportunities	.19	.02	.24	8.68	< .01	.50	1.99
Job resources	Social support-leaders	.22	.03	.24	8.43	< .01	.48	2.09
Job resources	Work autonomy	.05	.01	.15	6.41	< .01	.73	1.37
Job demands	Performance evaluation	-.10	.02	-.13	5.67	< .01	.71	1.41
Job demands	Student academic foundation	-.07	.02	-.07	3.16	< .01	.79	1.27
Job demands	Yearly teaching hours	-.03	.01	-.06	3.08	< .01	.89	1.12
Job resources	Social support-colleagues	.06	.02	.06	2.58	.01	.70	1.44
Job resources	Appraisal feedback guide	.04	.02	.06	2.35	.02	.63	1.59
R^2^ = .56; F = 112.01, *P* ≤ .05.								
**Impacting factors of physical/mental well-being**								
Job demands	Daily working hours	-.08	.01	-.19	6.76	< .01	.87	1.14
Job demands	Performance evaluation	-.17	0.03	-.19	5.90	< .01	.71	1.41
Job demands	Pressure of research work	-.06	.01	-.16	5.66	< .01	.83	1.21
Job resources	Training and development opportunities	.07	.04	.07	1.93	.05	.50	1.99
R^2^ = .20; F = 22.14, *P* ≤ .05.								
**Impacting factors of social well-being**								
Job resources	Social support-leaders	.43	.01	.56	36.97	< .01	.48	2.09
Job resources	Social support-colleagues	.31	.01	.38	30.53	< .01	.70	1.44
Job resources	Work autonomy	.03	.00	.09	6.91	< .01	.73	1.37
Job resources	Appraisal feedback guide	.03	.01	.06	4.28	< .01	.63	1.59
Job demands	Daily working hours	.01	.00	.05	4.45	< .01	.87	1.14
Job demands	Student academic foundation	-.03	.01	-.04	3.03	< .01	.79	1.27
Job demands	Yearly teaching hours	.01	0.00	.02	1.98	.05	.89	1.12
R^2^ = .87; F = 609.29, *P* ≤ .05.								

## Conclusions

We will make conclusions of this study in regard to the two research questions, based on what is found through the above analyses.

### Level and main characteristics of well-being

This study shows that language teachers in China’s private colleges have an intermediate level of overall well-being, which needs further improvement. Their well-being differs with demographic characteristics, especially age, teaching experience, and academic title. Teachers with larger or smaller ages enjoy higher well-being, while those with medium ages have lower well-being. Similarly, teachers with longer or shorter teaching experiences enjoy higher well-being and those with medium teaching experiences have lower well-being; teachers with higher or lower academic titles enjoy higher well-being and those with medium academic titles have lower well-being. There exists a “U-type” feature of teacher well-being with their age, teaching experience, and academic title. Their well-being shows no difference in respect to gender and education background.

### Impacting factors

In regard to the impacting factors of working environment on the well-being of teachers in private schools, we conclude what we examined in terms of job demands and job resources to echo the aforementioned hypotheses. As for job demands, performance evaluation, student academic foundation, and pressure of research work are the main negative impacting factors of teacher overall well-being in private schools, while class size, daily working hours, and yearly teaching hours have no significant impact. The first hypothesis H1 is partially validated. As for job resources, social support from leaders and colleagues, work autonomy, training and development opportunities, and appraisal feedback guide are key resources significantly impacting teacher overall well-being. The second hypothesis H2 is fully validated.

## Discussions

### Comparison with previous research

In regard to the impact of working environment on teacher well-being, there are few publications concerning about the group of teachers in private schools. A semi-structured interview with 8 teachers from private schools in Malta shows working hours, work climate (including relationships with the boss and colleagues) impact teacher well-being^10^. Most previous research on this topic concluded the impacting factors of working environment on teacher well-being in public schools, which we categorize as job demands and job resources (including what was found in the Maltese private schools) and form the two hypotheses in this study; hence we will mainly compare and discuss what we found in China’s private colleges with the job demands and job resources we categorized from previous research.

#### Job demands

Job demands, associated with energy depletion and psychological and/or physiological costs, are the risk factors which usually undermine teacher well-being^36^. This study finds that performance evaluation, student academic foundation, and pressure of research work are the main negative impacting factors of teacher overall well-being in private schools, and class size has no significant influence, which is coherent with previous research; while daily working hours and yearly teaching hours have no significant impact, which is different with previous findings. The same findings show these factors are universal and applying to teachers both in public and private schools. We will discuss the two different factors, i.e. daily working hours and yearly teaching hours, which impact teacher well-being in public schools but not for teachers in private schools.

We find that daily working hours and yearly teaching hours have no significant impact on overall well-being, and play different roles in some dimensions in this study. Daily working hours has negative impact only on physical/mental well-being, but slight positive impact on social well-being; while yearly teaching hours has negative impact only on subjective well-being, but positive impact on cognitive and social well-being. This may be attributed to the unique scenarios where full-time young teachers in China’s private colleges are usually supposed to participate in various faculty workshops for academic progress and take on huge amount of teaching work in the classroom staying with students. The more they exchange ideas and discuss problems, the more will they benefit and the better relationship will they have with other people. The negative and positive impact is offset, which results in no significant impact on overall well-being.

#### Job resources

This study finds that all the key resources concluded in previous research are also significant positive factors impacting teacher well-being in private schools. This is coherent for teachers from different types of schools, different locations, and different countries. It means job resources, like social support from leaders and colleagues, work autonomy, training and development opportunities, and appraisal feedback guide are major motivators to increase teacher well-being both in public schools and in private schools as well.

### Limitations

Teacher well-being is very complex and is impacted with a wide range of factors. This study is an attempt to explore the impact of working environment on the well-being of teachers in private schools. It still has limitations. First, teachers’ perceptions of well-being and working environment (job demands and job resources) are all self-reported in this study, but it could also be that a person’s level of well-being has an impact upon their perceptions of job demands and job resources; hence longitudinal studies should be conducted in the future to examine the possible bi-directional relationships. Second, we adopted the questionnaire by OECD, but reworded some vague items and deleted some items based on the scale analysis with ‘Cronbach α value if deleted’. The final questionnaire we used is slightly different from the original one by OECD, though it is tested to have good reliability and validity. Using such a measurement limits the ability to compare our results with samples from other studies. In the future, our questionnaire can be used with samples in other cultures around the world for comparison purposes, or with other samples of teacher groups within China.

### Generalizability

The samples of this study come from China’s private colleges, who came into being since late 1990s as a complement to public universities to meet the needs for those who failed in the college entrance examination and who could not afford the tuition fees to study abroad. Without the financial support from the government, their source of funds depends mainly on students’ tuition fees, from which most investors/sponsors also seek economic profits/rewards. The findings of this study may apply to similar private schools in terms of business mode, especially for promotion measures of teacher well-being in these schools.

## Implications

This study shows that there are some unique impacting factors of working environment on teacher well-being in private schools, which provides some implications for practice and policy considerations.

### Targeted management measures

Synthesizing all the influential factors, we can see that it is the school management that plays a key role in teacher well-being. Social support, work autonomy, training and teacher development, appraisal feedback and performance evaluation identified as significant impacting factors in this study are all subject to the leadership and management in a school. To promote teacher well-being, schools can take targeted measures which we discussed above to decrease job demands and increase job resources.

### Enough economic input

For private schools, enough economic input into running the school should be guaranteed. Some sponsors establish private schools as a business for economic profits, so they tend to reduce expenses to the lowest for the highest rewards. They are reluctant to recruit high level faculty with high salary and to organize high-quality training programs for the young teachers. Without just expenditure, the measures mentioned above to promote teacher well-being will become largely inefficient or come to naught.

### Democratic and professional educational administration

Patriarch-based management model is still employed in some private schools with excessive empowerment to the president, which results in low involvement of faculty in decision making and low efficiency of staff in working. Democracy is an essential component in schooling, and participatory democracy is one of the goals of modern school management transformation. Teachers should become masters of private schools and have a say in work decisions with enough autonomy. If the president is not an education expert, or even not an education professional, the school administration is even worse. Some sponsors serve concurrently as the school presidents, but they are not fully experienced in educational administration. School leaders of private schools are more important than national policies for the school management^37^, so it is crucial for them to be professional and expert in regard to school development and teacher well-being as well.

## Data Availability

The datasets generated during and/or analyzed during the current study are available from the corresponding author on reasonable request.
